# Global transcriptomic analysis identifies *SERPINE1* as a prognostic biomarker associated with epithelial-to-mesenchymal transition in gastric cancer

**DOI:** 10.7717/peerj.7091

**Published:** 2019-06-10

**Authors:** Bodong Xu, Zhigang Bai, Jie Yin, Zhongtao Zhang

**Affiliations:** 1 Department of General Surgery, Beijing Friendship Hospital, Capital Medical University, Beijing, China; 2 Key Laboratory of Cancer Invasion and Metastasis Research, National Clinical Research Center for Digestive Diseases, Beijing, China

**Keywords:** SERPINE1, Gastric cancer, Epithelial-to-mesenchymal transition, Bioinformatic analysis

## Abstract

**Background:**

The plasminogen activation system plays a pivotal role in regulating tumorigenesis. In this work, we aim to identify key regulators of plasminogen activation associated with tumorigenesis and explore potential mechanisms in gastric cancer (GC).

**Methods:**

Gene profiling datasets were extracted from the Gene Expression Omnibus (GEO) database. The differentially expressed genes (DEGs) were screened for and obtained by the GEO2R tool. The Database for Annotation, Visualization and Integrated Discovery was used for GO and KEGG enrichment analysis. Gene set enrichment analysis (GSEA) was performed to verify molecular signatures and pathways among The Cancer Genome Atlas or GEO datasets. Correlations between SERPINE1 and markers of epithelial-to-mesenchymal transition (EMT) were analyzed using the GEPIA database and quantitative real-time PCR (qRT-PCR). Interactive networks of selected genes were built by STRING and Cytoscape software. Finally, selected genes were verified with the Kaplan–Meier (KM) plotter database.

**Results:**

A total of 104 overlapped upregulated and 61 downregulated DEGs were obtained. Multiple GO and KEGG terms associated with the extracellular matrix were enriched among the DEGs. SERPINE1 was identified as the only regulator of angiogenesis and the plasminogen activator system among the DEGs. A high level of SERPINE1 was associated with a poor prognosis in GC. GSEA analysis showed a strong correlation between SERPINE1 and EMT, which was also confirmed with the GEPIA database and qRT-PCR validation. FN1, TIMP1, MMP2, and SPARC were correlated with SERPINE1.The KM plotter database showed that an overexpression of these genes correlated with a shorter survival time in GC patients.

**Conclusions:**

In conclusion, SERPINE1 is a potent biomarker associated with EMT and a poor prognosis in GC. Furthermore, FN1, TIMP1, MMP2, and SPARC are correlated with SERPINE1 and may serve as therapeutic targets in reversing EMT in GC.

## Introduction

Gastric cancer (GC) is one of the most lethal cancers with tremendous cancer-related mortality (Van Cutsem et al., 2016). For the last few decades, GC has generally been divided into histological subtypes according to the Lauren classification (Lauren, 1965). As medicine becomes more precise, there has been an urgent need for the use of molecular biology to more prominently guide the therapeutic strategies and target agents in GC. The Cancer Genome Atlas (TCGA) project had raised the molecular classification of four subtypes with distinct genetic signatures in GC (The Cancer Genome Atlas Research Network, 2014). Meanwhile, recent research has also proposed four GC subtypes on the basis of distinct molecular signatures (Cristescu et al., 2015).

The urokinase-type plasminogen activator (uPA) system plays a crucial role in tumorigenesis. By mediating proteolysis and the degradation of the extracellular matrix (ECM), the uPA system regulates tumor angiogenesis and locomotion via the activation of matrix metalloproteinases (MMPs) and latent growth factors (Santibanez et al., 2018). Plasminogen activator inhibitor-1 (PAI-1), encoded by the Serpin E1 (SERPINE1) gene, is a key regulator of the uPA system. PAI-1 is supposed to restrain the activation of uPA, whereas, paradoxical data implies that SERPINE1 may exert its role of promoting carcinogenesis in various cancers (Li et al., 2018). Emerging evidence indicates that SERPINE1 is upregulated in GC tissues compared with normal tissues. Elevated levels of SERPINE1 are significantly correlated with unfavorable clinical features and a poor prognosis in GC patients (Liao et al., 2018; Liu et al., 2018), suggesting a pro-tumor effect of SERPINE1 in gastric tumorigenesis.

The epithelial-to-mesenchymal transition (EMT), a shift from the epithelial to mesenchymal state with highly plastic and dynamic features, enables the cells to possess a greater ability for migration and invasion (Nieto et al., 2016). Previous reports demonstrated that the upregulation of SERPINE1 in TGFβ induced EMT in triple-negative breast cancer (Xu et al., 2018). The administration of exogenous recombinant PAI-1 promoted cell locomotion and increased the expression of EMT markers (Kang et al., 2016). However, it still remains unclear whether SERPINE1 is involved in EMT in gastric tumorigenesis.

In this study, we screened the differentially expressed genes (DEGs) associated with gastric tumorigenesis from the Gene Expression Omnibus (GEO) datasets. Through integrative analysis, we identified that SERPINE1 was correlated with EMT in GC and confirmed this with experimental validation. Further investigations revealed that key regulators of the EMT process cooperated with SERPINE1 in GC.

## Materials and Methods

### Extraction of oligonucleotide microarrays and RNA sequencing data of gastric cancer

Three transcriptional profiling datasets of GC were downloaded and extracted from the GEO database. The GSE54129 dataset was composed of 111 GC and 21 normal tissues and was performed on the Affymetrix Human Genome U133 Plus 2.0 Array platform. The GSE63089 dataset contained 45 paired GC and normal tissues and was performed on the Affymetrix Human Exon 1.0 Array platform (Zhang et al., 2015). The GSE65801 dataset contained 32 paired GC and noncancerous tissues and was performed on the Agilent Human GE 8x60K Microarray platform (Li et al., 2015). Gene symbols were mapped to the probes according to the annotation profile of each platform. Data of the expression matrix, genetic alterations, and the clinical features of patients in TCGA STAD were downloaded from the cBioPortal database (https://www.cbioportal.org). All above-mentioned profiling datasets were accessible online without any conflicts or limitations.

### Screening and bioinformatic annotations of DEGs in gastric cancer

The web-accessible analysis tool GEO2R was utilized on its default settings to screen the DEGs of each dataset. The cut-off value for the filtration criteria was set at adjusted *p* < 0.05, logFC ≥ 1. The DEGs for subsequent GO and KEGG analysis were obtained by the overlap of filtered genes in each dataset via an online Venn diagram tool (http://bioinformatics.psb.ugent.be/webtools/Venn/). The DAVID database (https://david.ncifcrf.gov/tools.jsp) was used for GO and KEGG analysis (Huang, Sherman & Lempicki, 2009a, 2009b). Enriched GO and KEGG terms with *p-*value < 0.05 were considered as statistical significance. Histograms of enriched GO terms and bubble plots of KEGG pathway enrichment were implemented with the ggplot2 package (https://ggplot2.tidyverse.org/) in an R language environment.

### Gene set enrichment analysis

Gene set enrichment analysis was carried out as previously described (Subramanian et al., 2005). Defined gene sets, including angiogenesis (HALLMARK_ANGIOGENESIS, M5944), EMT (HALL_EPITHELIAL_MESENCHYMAL_TRANSITION, M5930/JECHLINGER_EPITHELIAL_TO_MESENCHYMAL_TRANSITION_UP, M1406/GOTZMANN_EPITHELIAL_TO_MESENCHYMAL_TRANSITION_UP, M1373), uPA and uPAR induced pathways (PID_UPA_UPAR_PATHWAY, M174), and TGFβ signaling pathway (KEGG_TGF_BETA_SIGNALING_PATHWAY, M2642 / TGFB_UP.V1_UP, M2839), were obtained from the molecular signatures database (http://software.broadinstitute.org/gsea/msigdb) of Broad Institute. Analysis was performed with default settings and a false discovery rate (FDR) < 0.25 was considered as statistical significance.

### Cell culture and reagents

The MGC803 and AGS GC cell lines were purchased from the Cell Center of Peking Union Medical College. PCR and cell culturing verified that the cell lines were free of mycoplasma contamination. Authentication of the cell lines was confirmed by STR profiling. All of the results can be viewed on the National Infrastructure of Cell Line Resource website (http://cellresource.cn). Cells were cultured in DMEM or RPMI-1640 medium (Gibco, Carlsbad, CA, USA) with 10% fetal bovine serum (Gibco, Carlsbad, CA, USA). Cells were cultured with 5% CO_2_ at 37 °C. Tiplaxtinin (PAI-039) was purchased from Selleck (Houston, TX, USA).

### Total RNA extraction and qRT-PCR validation

Cells were digested and planted in six-well plates (1 × 10^6^ cells per well). The culture medium was replaced with the administration of different concentrations of Tiplaxtinin after cell adherence. Cells were incubated for 12 h. Total RNA was extracted by the TRIzol (Invitrogen, Carlsbad, CA, USA) reagent and the cDNA was synthesized using the iScript™ Synthesis Kit (BioRad, Hercules, CA, USA) following the manual’s instructions. The PCR procedure was performed by PowerUp™ SYBR™ Green Master Mix Kit (Applied Biosystems™, Foster City, CA, USA). GAPDH was used as an endogenous control. The 2^−ΔΔCt^ method was used to calculate the relative expression of each gene. Each sample per reaction was performed in triplicate. The sequence of primers is listed in Table 1. The primer of GAPDH was purchased from Sangon Biotech (Shanghai, China).

**Table 1:** Primers of quantitative real-time polymerase chain reaction.

Gene symbol	Primers	Sequence
TWIST1	Forward	GTACATCGACTTCCTCTACCAG
	Reverse	CATCCTCCAGACCGAGAAG
TWIST2	Forward	CTCTGACAAGCTGAGCAAGATC
	Reverse	AGCTGGTCATCTTATTGTCCAT
CDH2	Forward	CGATAAGGATCAACCCCATACA
	Reverse	TTCAAAGTCGATTGGTTTGACC
FN1	Forward	AATAGATGCAACGATCAGGACA
	Reverse	GCAGGTTTCCTCGATTATCCTT

### Construction of protein-protein interaction network involved in EMT

To further investigate the sub-clusters of DEGs involved in EMT, overlapped genes between DEGs of GC and a hallmark gene set of EMT were selected to construct the protein-protein interaction (PPI) network by utilizing the STRING database (https://string-db.org) (Szklarczyk et al., 2017). To enhance the dependability of the PPI network, the highest confidence (>0.900) was chosen as the required interaction score. The visualization of the interaction network was implemented via Cytoscape software v3.5.

### Validation of prognostic values of EMT-related genes in GEO datasets

The web-based Kaplan–Meier (KM) plotter database is capable of assessing specific genes of survival using the data of 1,065 GC patients from the GEO database (Szasz et al., 2016). Overall survival (OS) and first progression (FP) were selected as the main indicators of prognostic assessment. Mean expression of all probes of the same gene was calculated as the expression of each gene. The log-rank *p* < 0.05 was considered as statistical significance.

### Statistical analysis

Analysis of the receiver operator characteristic (ROC) curves was performed to explore the efficacy of SERPINE1 in discriminating different molecular subtypes (EMT and non-EMT subtype) and OS prognosis (good OS ≥2 years, living and poor OS <1 year, deceased) in GC. The KM curves were carried out to compare the survival distributions between patients with high and low mRNA levels of SERPINE1 in the TCGA STAD dataset. Univariate and multivariate Cox regressions were implemented to investigate the prognostic impact of SERPINE1 in GC patients of TCGA STAD dataset. Pearson correlation tests were used to assess the relationship between SERPINE1 and EMT-related genes in the TCGA STAD dataset via the GEPIA database (Tang et al., 2017). An independent sample *t-*test and one-way ANOVA was used to compare the statistical significance between two or more samples, respectively. Two-tailed *p-*values < 0.05 were considered as statistically significance. Statistical analysis was performed using the SPSS 19.0 (Chicago, IL, USA) and Graphpad Prism 5.0 software (Graphpad Software, Inc., San Diego, CA, USA).

## Results

### Screening and bioinformatic analysis of DEGs in GEO datasets

**Figure 1:**
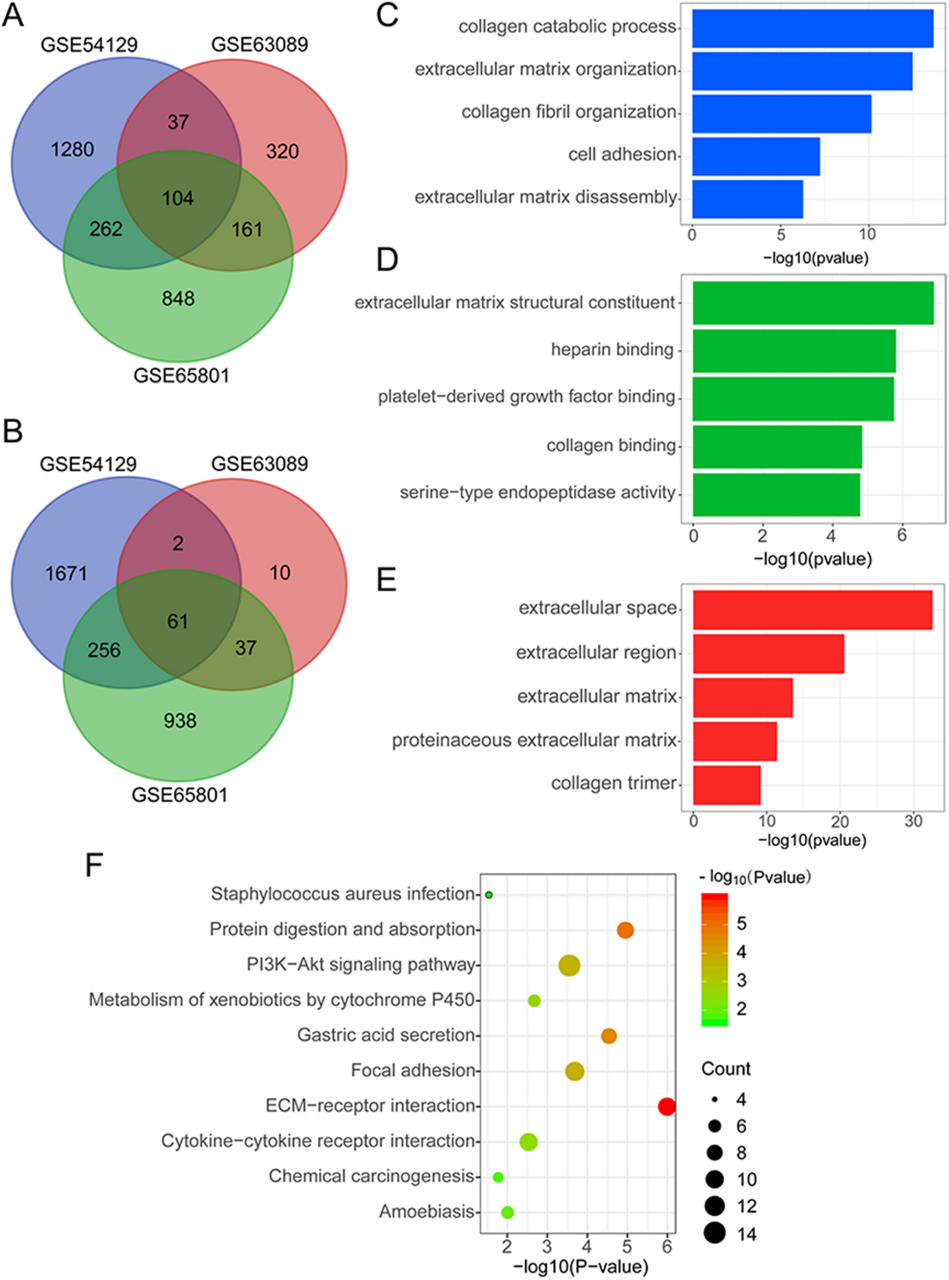
Screening and bioinformatic analysis of DEGs in GEO datasets. (A) The venn graph of up-regulated DEGs in the GSE54129, GSE63089, and GSE65801 datasets. (B) The venn graph of down-regulated DEGs in the GSE54129, GSE63089, and GSE65801 datasets. The gene ontology analysis of DEGs, including (C) biological process (D) molecular function and (E) cellular component. (F) The KEGG enrichment analysis of DEGs in the GSE54129, GSE63089, and GSE65801 datasets.

To identify DEGs associated with gastric tumorigenesis, we applied the GEO2R tool to screen for DEGs between normal and tumor tissues. To enhance the dependability of our data, we overlapped DEGs from each GEO dataset to obtain commonly upregulated and downregulated DEGs. A total of 104 overlapped upregulated and 61 downregulated DEGs were identified among multiple datasets (Figs. 1A and 1B). To further investigate the functions of filtered DEGs, we employed GO and KEGG enrichment analysis to annotate dysregulated genes and signaling pathways. The results of the GO analysis showed that biological processes such as collagen catabolic process, ECM organization, and collagen fibril organization, and molecular functions like ECM structural constituent, heparin binding, and platelet-derived growth factor binding were found to be significantly enriched among DEGs (Figs. 1C and 1D). Analysis of the cellular components indicated that proteins encoded by DEGs were mainly located in the extracellular space and regions (Fig. 1E). KEGG enrichment analysis showed that ECM-receptor interaction, protein digestion and absorption, PI3K-AKT signaling pathway, gastric acid secretion, and focal adhesion were significantly enriched in DEGs (Fig. 1F), suggesting that alterations of the ECM might be responsible for the malignant progress of GC.

### Overexpression of SERPINE1 in gastric cancer is identified by integrative approach

**Figure 2:**
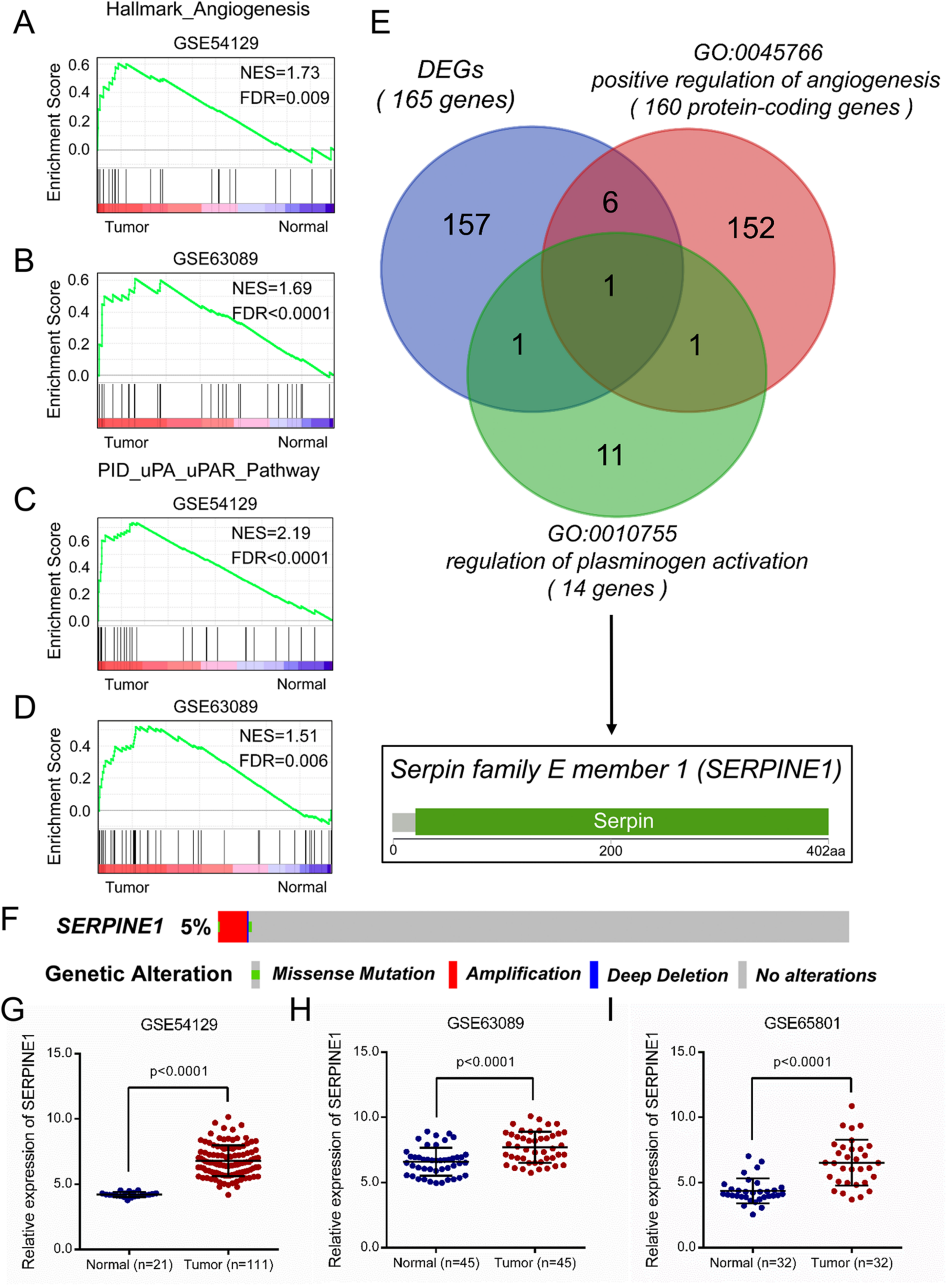
Overexpression of SERPINE1 in gastric cancer is identified by integrative approach. The GSEA analysis between normal gastric mucosa and gastric cancer tissues using the hallmark gene set of angiogenesis in (A) GSE54129 and (B) GSE63089 datasets. The GSEA analysis between normal gastric mucosa and gastric cancer tissues using gene set of uPA pathway in (C) GSE54129 and (D) GSE63089 datasets. NES, normalized enrichment score. FDR, false discovery rate. (E) The venn graph of DEGs and GO terms (positive regulation of angiogenesis and regulation of plasminogen activation). (F) The genetic alterations (mutation and copy number) of SERPINE1 in TCGA STAD dataset. STAD, stomach adenocarcinoma. The relative mRNA level of SERPINE1 in normal and gastric cancer tissues of the (G) GSE54129, (H) GSE63089, and (I) GSE65801 datasets were analyzed using the independent sample *t*-test, respectively.

During tumorigenesis, the angiogenic regulators remain activated to support tumor growth (Ferrara, 2009). As a critical part of fibrinolytic system, the uPA system is well known in regulating tissue remodeling, angiogenesis, and cell mobility (Su et al., 2016). We checked if angiogenesis and uPA pathways were activated in GC. As we expected, GSEA analysis showed that gene sets of hallmark angiogenesis and the uPA pathway were significantly enriched in GC compared with normal tissues (Figs. 2A–2D). To further identify key regulators in the above-mentioned processes, we screened overlapped genes between filtered DEGs and genes involved with the positive regulation of angiogenesis and plasminogen activation. Interestingly, SERPINE1 remained to be the only overlapped gene among three gene lists (Fig. 2E). To exclude the impact of genetic variations on the mRNA level of SERPINE1, we checked the genetic alterations of SERPINE1 in the TCGA STAD dataset. Results indicated that only 5% of GC patients showed genetic alterations, including missense mutation, amplification, and deep deletion (Fig. 2F), which suggested the minimal influence of genetic variations on SERPINE1 expression. Levels of SERPINE1 were significantly increased in GC tissues compared with normal issues (Figs. 2G–2I). Altogether, we confirmed that SERPINE1, a key regulator of angiogenesis and the uPA system, was remarkably overexpressed in GC.

### Elevated mRNA level of SERPINE1 predicts poor outcome of gastric cancer patients

**Figure 3:**
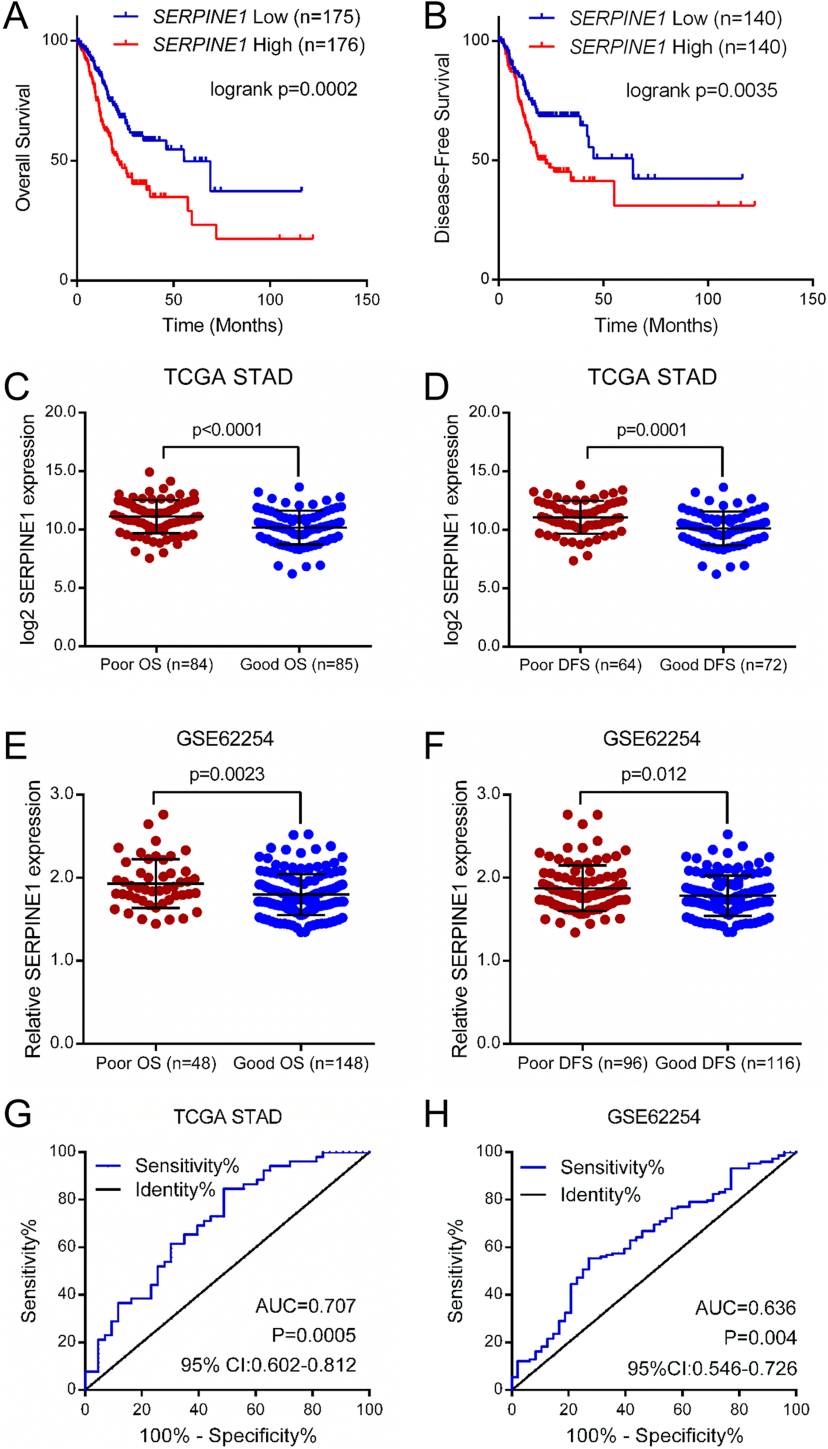
Elevated mRNA level of SERPINE1 predicts poor outcome of gastric cancer patients. The KM curves of (A) OS and (B) DFS in TCGA STAD cohort. The GC patients were divided into two groups by the median expression value of SERPINE1. The expression of SERPINE1 in TCGA STAD cohort with poor and good (C) OS and (D) DFS were analyzed using the independent sample *t*-test, respectively. The expression of SERPINE1 in GSE62254 cohort with poor and good (E) OS and (F) DFS were analyzed using the independent sample *t*-test, respectively. OS, overall survival. DFS, disease free survival. The ROC curves of SERPINE1 mRNA level in discriminating GC patients with poor and good OS in (G) TCGA STAD and (H) GSE62254 cohorts. ROC, receiver operator characteristic; AUC, area under roc curve; 95% CI, 95% confidence interval.

To investigate the prognostic value of mRNA levels of SERPINE1 in GC patients, KM curves were plotted in GC patients with high or low expression groups. Results showed that patients with higher mRNA levels of SERPINE1 had a shorter OS and disease-free survival (DFS) in GC (Figs. 3A and 3B), suggesting that the higher expression of SERPINE1 was a risk factor for the prognosis of GC. Univariate and multivariate analysis revealed a higher level of SERPINE1 as an independent predictor for poor prognosis in GC patients (Table 2). To verify our survival analysis, pre-defined poor (<1 year, deceased) and good (>2 years, living) OS groups of GC patients in the TCGA STAD and GSE62254 datasets were selected for further study. Results indicated that the level of SERPINE1 were significantly higher in patients with a poor OS vs. those with a good OS. Similar results were also observed in patients with poor (<1 year, recurred or deceased) and good (>2 years, disease free and living) DFS (Figs. 3D–3F). Moreover, ROC analysis indicated that the mRNA level of SERPINE1 was able to discriminate a poor and good OS in GC patients (Figs. 3G and 3H). Taken together, a higher mRNA level of SERPINE1 was correlated with a poor prognosis in GC patients.

**Table 2:** Univariate and multivariate analysis of SERPINE1 mRNA level and survival in TCGA STAD dataset.

Variable	Univariate analysis			Multivariate analysis^3^		
	HR^1^	95%CI^2^	*p*-value	HR	95%CI	*p*-value
Overall survival (*n* = 351)						
Gender						
Male (*n* = 220)	1.325	0.924–1.900	0.125			
Female (*n* = 131)						
Age (years)						
>60 (*n* = 234)	1.731	1.182–2.533	0.005	2.076	1.407–3.062	0.000
≤60 (*n* = 117)						
T stage						
T3/T4 (*n* = 263)	1.715	1.109–2.652	0.015	1.198	0.720–1.994	0.486
T1/T2 (*n* = 88)						
N stage						
N1/2/3 (*n* = 241)	1.906	1.259–2.885	0.002	1.405	0.797–2.478	0.240
N0 (*n* = 110)						
M stage						
M1 (*n* = 24)	1.945	1.074–3.523	0.028	1.993	1.073–3.702	0.029
M0 (*n* = 327)						
TNM stage						
Stage III/IV (*n* = 193)	1.944	1.359–2.779	0.000	1.325	0.764–2.298	0.316
Stage I/II (*n* = 158)						
G grade						
G3 (*n* = 226)	1.434	1.002–2.052	0.049	1.452	1.006–2.094	0.046
G1/G2 (*n* = 125)						
SERPINE1						
High (*n* = 176)	1.941	1.377–2.737	0.000	1.843	1.305–2.603	0.001
Low (*n* = 175)						
Disease-free survival (*n* = 280)						
Gender						
Male (*n* = 178)	2.179	1.357–3.497	0.001	2.021	1.256–3.252	0.004
Female (*n* = 102)						
Age (years)						
>60 (*n* = 175)	0.999	0.670–1.490	0.996			
≤60 (*n* = 105)						
T stage						
T3/T4 (*n* = 204)	1.408	0.882–2.247	0.151			
T1/T2 (*n* = 76)						
N stage						
N1/2/3 (*n* = 182)	1.774	1.121–2.807	0.014	1.658	0.925–2.973	0.089
N0 (*n* = 98)						
M stage						
M1 (*n* = 16)	1.482	0.647–3.393	0.352			
M0 (*n* = 264)						
TNM stage						
Stage III/IV (*n* = 142)	1.500	1.007–2.234	0.046	1.030	0.620–1.711	0.908
Stage I/II (*n* = 138)						
G grade						
G3 (*n* = 176)	1.198	0.795–1.805	0.388			
G1/G2 (*n* = 104)						
SERPINE1						
High (*n* = 140)	1.800	1.206–2.687	0.004	1.755	1.175–2.621	0.006
Low (*n* = 140)						

**Notes:**

1 Hazard ratio.

2 Confidence interval of the HR.

3 Multivariate analysis of SERPINE1 was adjusted for included data like T, N, M stages, G grades, age or gender.

### Overexpression of SERPINE1 is correlated with EMT in gastric cancer

**Figure 4:**
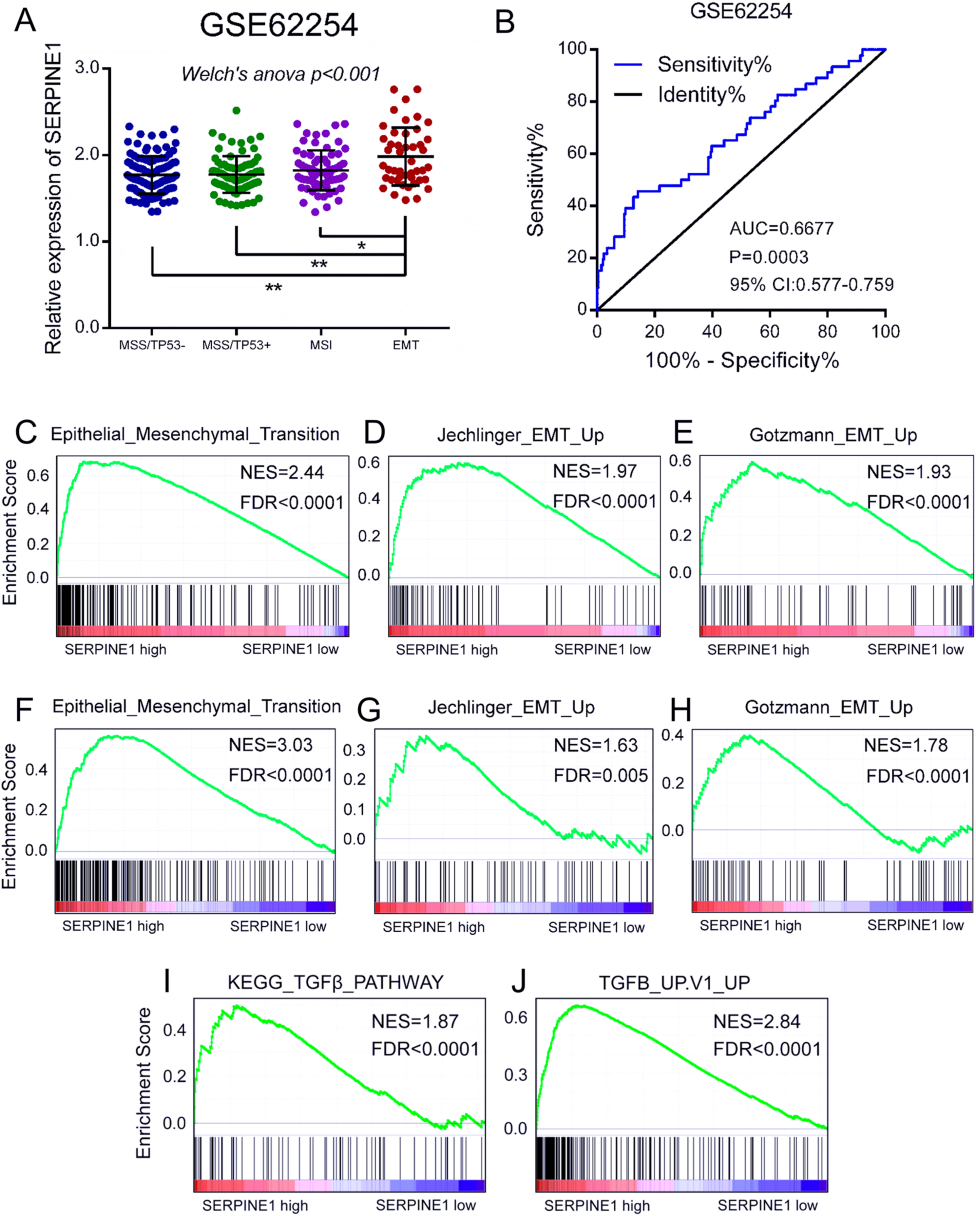
Overexpression of SERPINE1 is correlated with EMT in gastric cancer. (A) The mRNA level of SERPINE1 among the molecular subtypes of MSS/TP53−, MSS/TP53+, MSI, and EMT in the GSE62254 dataset was analyzed using the Welch’s ANOVA. MSS, microsatellite stability; MSI, microsatellite instability; EMT, epithelial to mesenchymal transition. (B) The ROC curve of SERPINE1 in discriminating EMT and non-EMT molecular subtypes of GSE62254 dataset. The GSEA validation of (C–E) GSE63089 dataset and (F–H) TCGA STAD dataset using the EMT-related gene sets. (I and J) The GSEA analysis of the TCGA STAD dataset using the gene sets of TGFβ signaling pathway. NES, normalized enrichment score. FDR, false discovery rate.

Previous reports identified four molecular subtypes associated with distinct clinical outcomes in GC (Cristescu et al., 2015). To further investigate the possible mechanisms that SERPINE1 may involve in GC, we compared the mRNA level of SERPINE1 among four subtypes including MSS/TP53 activation, MSS/TP53 loss, microsatellite instability (MSI), and EMT. Interestingly, the mRNA level of SERPINE1 was much higher in the EMT subtype than in other subtypes, indicating a potential correlation between SERPINE1 and EMT in GC (Fig. 4A). ROC analysis showed that the mRNA level of SERPINE1 effectively discriminated between EMT and non-EMT subtypes in the GSE62254 dataset (Fig. 4B). Furthermore, GSEA analysis demonstrated that EMT-related gene sets were significantly enriched in patients with higher SERPINE1 expression in the GSE63089 and TCGA STAD datasets (Figs. 4C–4H). TGFβ exerts as a master inducer of EMT in various cancers. To determine whether SERPINE1 was involved in the TGFβ-induced signaling pathway, we employed TGFβ associated gene sets to perform GSEA analysis in the TCGA dataset. Results showed that a high SERPINE1 expression was significantly correlated with the activation of the TGFβ signaling pathway (Figs. 4I and 4J). Above all, the expression of SERPINE1 was significantly upregulated in the EMT subtype of GC patients and a higher expression of SERPINE1 was positively correlated with TGFβ-induced EMT in GC.

### Correlation and validation of EMT markers with SERPINE1 in gastric cancer

**Figure 5:**
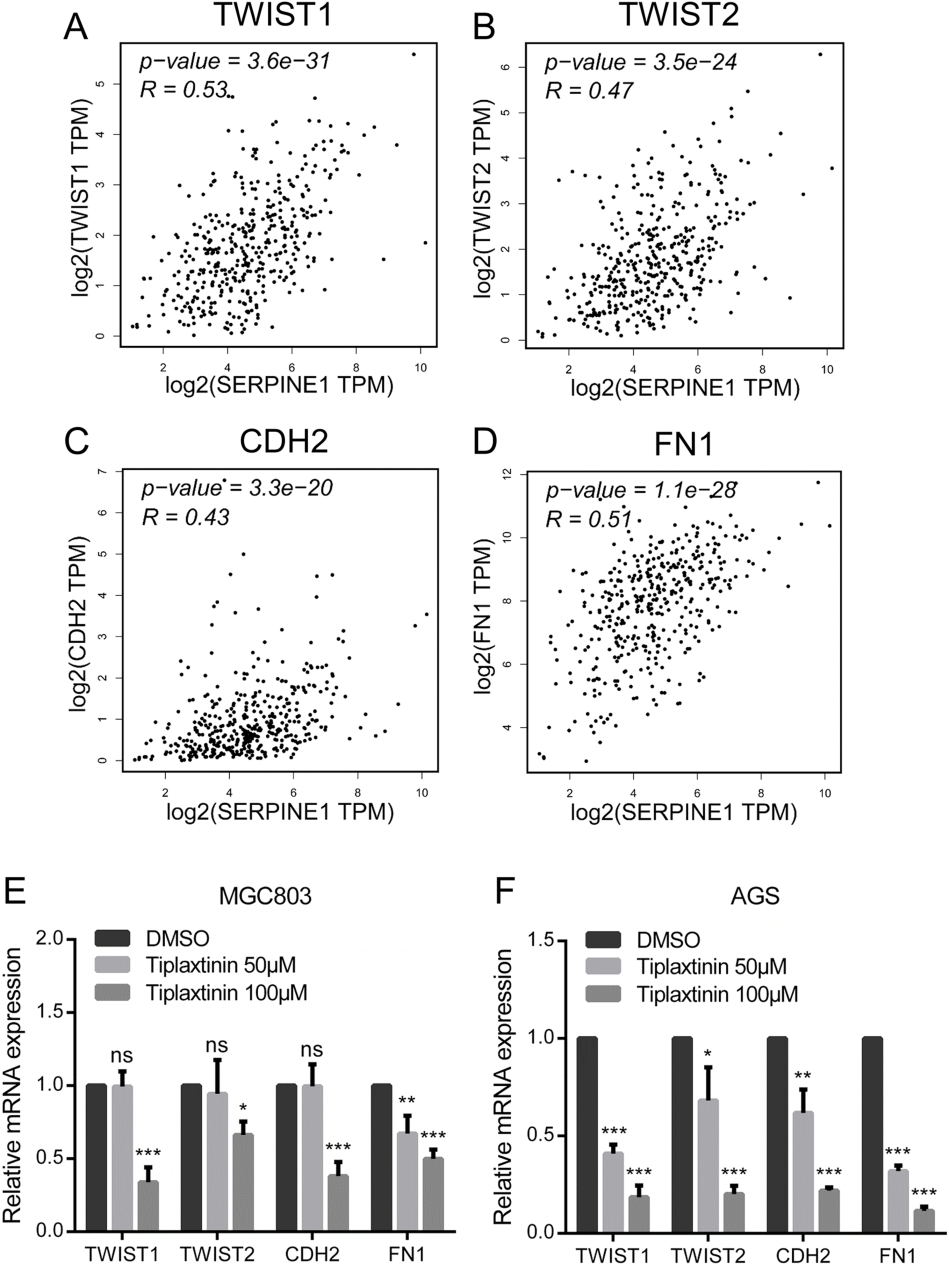
Correlations and validations of EMT markers with SERPINE1 in gastric cancer. The pearson correlations between SERPINE1 and the EMT markers, including (A) TWIST1, (B) TWIST2, (C) CDH2, and (D) FN1, in the TCGA STAD dataset. The gastric cancer (E) MGC803 and (F) AGS cells were treated with 50 and 100 μM Tiplaxtinin for 12 h. The mRNA expression of TWIST1, TWIST2, CDH2, and FN1 were examined by qRT-PCR. The gene expression was normalized to GAPDH. *p < 0.05, **p < 0.01, ***p < 0.001; ns, no statistical significance. Data were mean ± SD. Data were the representatives of three independent experiments.

To explore the regulatory mechanism between SERPINE1 and EMT in GC, correlations between the expression of SERPINE1 and the EMT markers were analyzed using the GEPIA database. As expected, the expression of SERPINE1 was positively correlated with EMT markers (TWIST1, TWIST2, CDH2, and FN1) in the TCGA STAD dataset (Figs. 5A–5D). To further verify our bioinformatic analysis, Tiplaxtinin, a specific inhibitor of SERPINE1, was selected for biological validation. The mRNA expression of the EMT markers significantly decreased following treatment with Tiplaxtinin in a concentration-dependent manner (Figs. 5E and 5F), indicating that the inhibition of SERPINE1 sufficiently suppressed the expression of EMT markers in GC.

### Identification of EMT-related genes associated with SERPINE1 in gastric cancer

**Figure 6:**
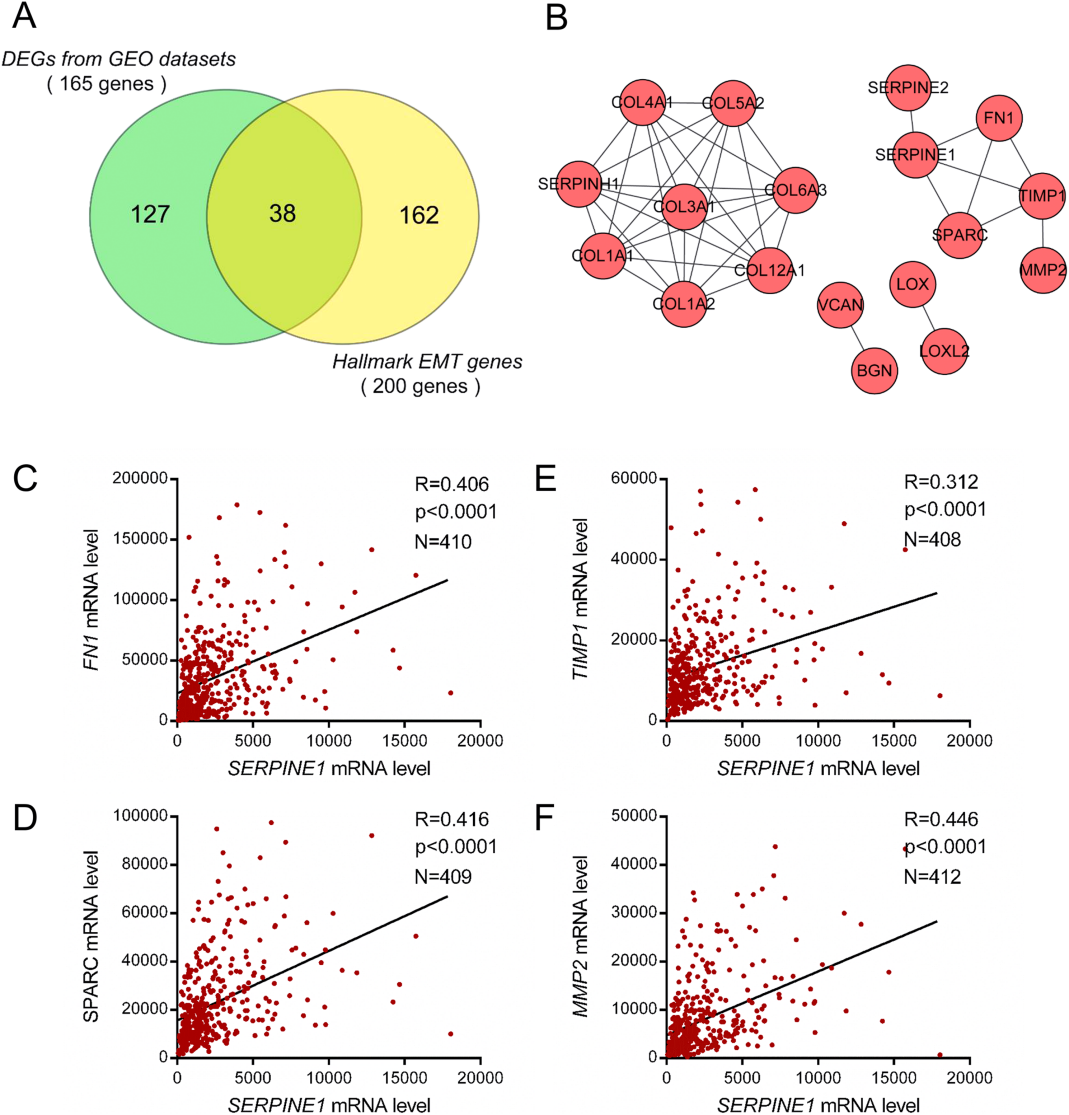
Identification of EMT-related genes associated with SERPINE1 in gastric cancer. (A) The venn graph of overlapped DEGs from the GEO datasets and hallmark EMT genes. (B) The PPI network of the EMT-related genes of DEGs from GEO datasets. The pearson correlation analysis of mRNA level of SERPINE1 with the EMT-related genes in the common cluster including (C) FN1, (D) SPARC, (E) TIMP1, and (F) MMP2 were analyzed among patients in the TCGA STAD dataset.

To explore the potential molecules and underlying mechanisms linked to SERPINE1, we obtained 38 EMT-related genes among DEGs from the intersection of two gene lists (Fig. 6A). A PPI network of these 38 EMT genes was constructed to reveal the potential interactions using the STRING database. Four gene clusters were identified among 38 EMT-related DEGs according to the highest confidence. Among these clusters, we noticed the genes including SERPINE1, SERPINE2, FN1, SPARC, TIMP1, and MMP2 were closely connected in a common cluster (Fig. 6B), suggesting a potential regulatory network among these molecules. To further validate our data, we performed a Pearson correlation analysis between SERPINE1 and these genes in the TCGA STAD dataset. As we expected, genes including FN1, SPARC, TIMP1, and MMP2 showed high correlation coefficients (*R* > 0.3) with SERPINE1 in GC (Figs. 6C–6F), suggesting potential regulations between SERPINE1 and these EMT-related genes.

### Prognostic values of EMT-related genes correlated with SERPINE1 in gastric cancer patients

**Figure 7:**
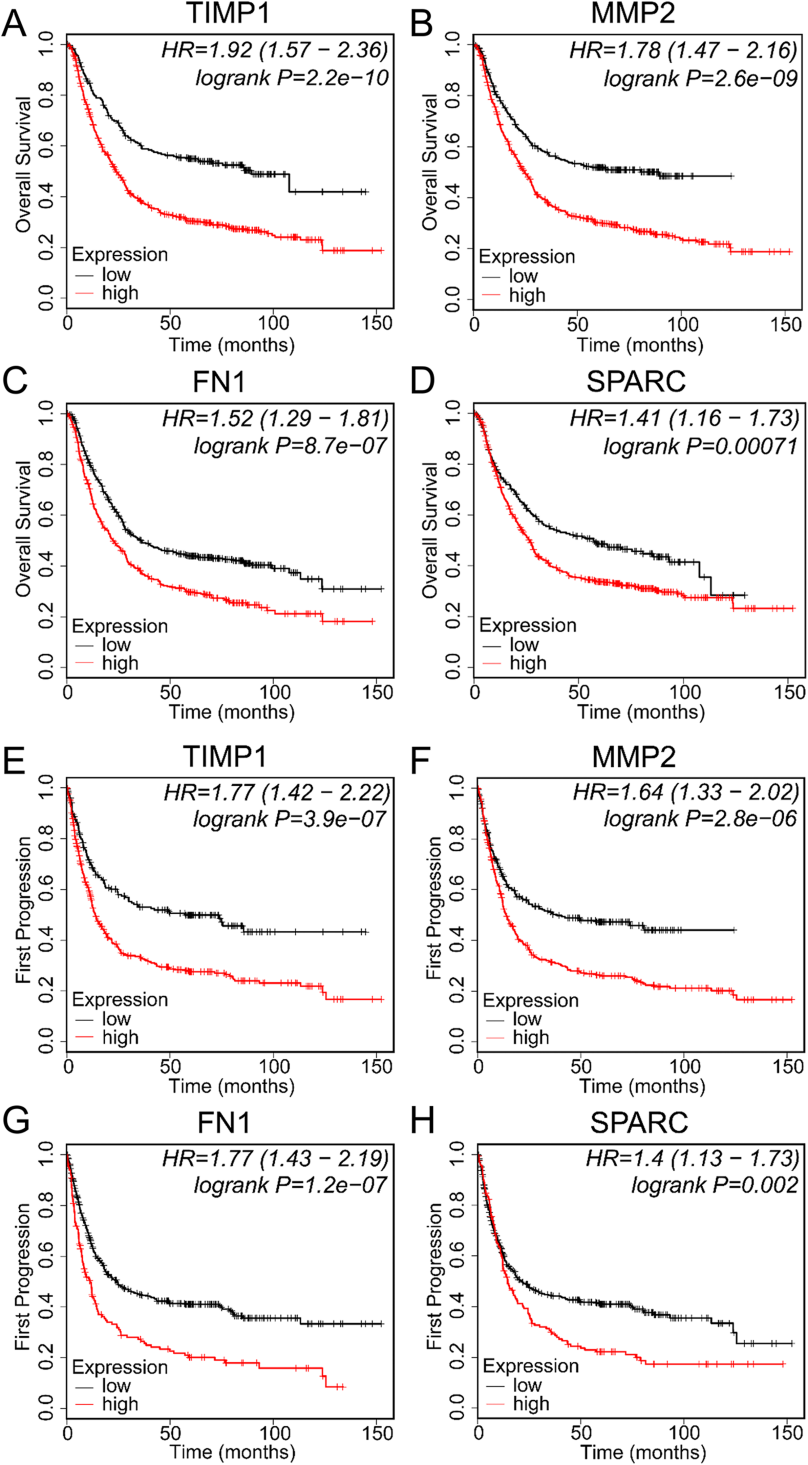
Prognostic values of EMT-related genes correlated with SERPINE1 in gastric cancer patients. The KM curves of EMT-related genes, including (A) TIMP1, (B) MMP2, (C) FN1, and (D) SPARC, for OS in gastric cancer patients. The KM curves of EMT-related genes, including (E) TIMP1, (F) MMP2, (G) FN1, and (H) SPARC, for FP in gastric cancer patients. OS, overall survival; FP, first progression.

To access the prognostic values of genes tightly correlated with SERPINE1, we performed a KM curve for each gene (TIMP1, MMP2, FN1, and SPARC). Similar to those of SERPINE1, the results showed that a higher expression of TIMP1, MMP2, FN1, and SPARC was significantly correlated with shorter OS and FP in GC patients (Fig. 7).

## Discussion

In this study, we screened multiple GEO datasets and obtained 104 overlapped upregulated and 61 downregulated DEGs. GO annotations recognized the functional enrichment of DEGs in the collagen catabolic process, ECM organization, and cell adhesion. KEGG analysis showed enrichment of ECM-receptor interaction, protein digestion and absorption, PI3K-Akt signaling pathway and focal adhesion, which indicated that the alteration of ECM was a hallmark of GC. We identified that the angiogenesis related gene SERPINE1, a key regulator of the uPA system, was overexpressed in GC. Further investigations demonstrated that a higher mRNA level of SERPINE1 predicted poor clinical prognosis in GC patients in the TCGA STAD and GEO datasets. Moreover, we verified the strong correlation between the SERPINE1 and EMT process in GC by integrative analysis and dependable validation. The constructed PPI network revealed that EMT-related genes (FN1, MMP2, TIMP1, and SPARC) were closely linked with SERPINE1, which developed a potential regulatory network of SERPINE1 in GC. Pearson correlation analysis showed positive correlations between mRNA levels of SERPINE1 and EMT related genes. KM survival analysis indicated that the four-gene cluster of EMT associated with SERPINE1 was correlated with a poor outcome in GC, which provided a more comprehensive insight into SERPINE1 and might serve as the therapeutic target in reversing EMT of GC.

The uPA system is mainly composed of four molecules including urokinase (encoded by PLAU gene), urokinase receptor (encoded by PLAUR gene), PAI-1 (encoded by SERPINE1 gene) and PAI-2 (encoded by SERPINB2 gene). In cancers, the uPA system catalyzes plasminogen to plasmin and directly induces the degradation of the ECM components, which leads to the activation of metalloproteinases and the release of latent growth factors (Laufs, Schumacher & Allgayer, 2006). PAI-1, which belongs to the family of serine proteinase inhibitors, is the main suppressor of uPA (Kruithof, Baker & Bunn, 1995). Previous reports have revealed that PAI-1 is a key regulator of angiogenesis. The physiological concentration of PAI-1 was necessary for tumor invasion and vascularization, while an excessive or low level of PAI-1 both demonstrated the suppression of tumorigenesis (Bajou et al., 2004; Kwaan, Mazar & McMahon, 2013). HUVECs showed impaired angiogenic potential when cultured with a conditional media from SERPINE1^KD^ cells (Gomes-Giacoia et al., 2013). A higher level of SERPINE1 was correlated with the angiogenic pattern in NSCLC patients (Offersen et al., 2007). An association between SERPINE1 and vessel remodeling was also observed in breast cancer (Fox et al., 2001). In this study, we confirmed the abnormal activation of angiogenesis and the uPA/uPAR pathway in GC by GSEA analysis. Through the overlap of genes involved in angiogenesis and plasminogen activation, we identified that SERPINE1 was the only key regulator of these processes in GC.

The overexpression of SERPINE1 was observed in many tumors, including GC. The higher expression of SERPINE1 was related with an unfavorable prognosis (Pavon et al., 2015). In our study, the overexpression of SERPINE1 was observed in GC tissue, and high mRNA levels of SERPINE1 was correlated with a poor clinical prognosis in GC patients. It was reported that the elevated expression of SERPINE1 was correlated with more aggressive lymph node metastasis based on a DNA microarray and TMA analysis in GC (Suh et al., 2015). A meta-analysis indicated that SERPINE1 expression was significantly associated with advanced T/N stage, and lymphatic and vascular invasion in gastroesophageal cancer (Brungs et al., 2017). Recently, studies based on a public database showed that SERPINE1 was upregulated in GC and correlated with poor outcomes in GC patients (Liao et al., 2018; Liu et al., 2018). Similarly, the authors identified overlapped DEGs and enriched functions among multiple datasets. Then, SERPINE1 was recognized as a potential marker of the prognosis for GC patients by integrative analysis of the constructed PPI network of filtered DEGs. However, whether SERPINE1 potentially participated with or was involved in any biological processes was not reported. At present, we found that the expression of SERPINE1 was much higher in the EMT subtype compared with the non-EMT subtypes, and confirmed the evidence between SERPINE1 and EMT in GC via GSEA analysis. Moreover, the overexpression of SERPINE1 promoted the EMT-mediated metastasis by the activation of STAT3 signaling in NSCLC cells (Lin et al., 2017). The enhanced proliferation and migration of breast cancer cells was induced by recombinant PAI-1 while the administration of Tiplaxtinin significantly reversed the malignant phenotype (Fang et al., 2018). Induced SERPINE1 by TGFβ stimulation increased expression of EMT markers, including N-cadherin, Snail and Twist (Xu et al., 2018). As a target gene of TGFβ signaling, SERPINE1 was correlated with TGFβ activity in multiple cancers (Frei et al., 2015; Sitaram et al., 2016). In our study, Tiplaxtinin significantly repressed the expression of EMT markers, which was consistent with previous reports. Furthermore, we verified the correlation between the SERPINE1 and TGFβ-induced signaling pathway by GSEA analysis. However, we did not validate the regulatory relationship and potential mechanism between SERPINE1 and TGFβ-induced EMT under experimental condition, which was one of the limitations in this study.

Cellular processes always require functional associations among various proteins and molecules. For filtered DEGs, specific constructed PPI networks are conducive for better insight into possible interactions and mechanisms. In this study, we identified a gene cluster (SERPINE1, FN1, TIMP1, SPARC, and MMP2) of EMT in GC. A higher expression of fibronectin was observed in multiple cancers and correlated with malignancy and poor outcomes (Gao et al., 2017; Yi et al., 2016). A previous study showed that SERPINE1 promoted fibronectin assembly in a uPA-independent manner (Vial & McKeown-Longo, 2008). Elevated TIMP1 was observed in GC and associated with a more aggressive phenotype (De Mingo et al., 2007; Omar et al., 2018; Seo et al., 2007). SPARC was reported to be involved in tumorigenesis and EMT of multiple cancers (Chang et al., 2017; Fenouille et al., 2012). The upregulation of SPARC was associated with tumor metastasis and poor prognosis in GC (Li et al., 2016; Sato et al., 2013; Wang et al., 2004; Zhao et al., 2010). The level of MMP2 was significantly higher in tumor tissues and correlated with unfavorable clinical features and prognosis in GC (Alakus et al., 2008; Zhang et al., 2011; Zheng et al., 2006). Consistent with a recent report (Yan et al., 2018), we confirmed that the expression of FN1, TIMP1, SPARC, and MMP2 was associated with a poor prognosis in GC. Furthermore, we uncovered the significant correlations and potential interactions between SERPINE1 and these four genes in GC. However, more investigations need to be carried out to validate the function and regulation between SERPINE1 and EMT-related genes in GC.

In this study, we identified that SERPINE1 was overexpressed and associated with EMT in GC using an integrative bioinformatics approach and dependable experimental validation. However, there were a few limitations in our study. First, the suppression of SERPINE1 by a specific inhibitor might differ depending on the manipulation methods like RNA interference, which might lead to different results affecting EMT-related genes in GC cells. Secondly, intricate modifications of biological processes and methodological variations of microarrays and RNA sequencing data might cause deviations and differences of our bioinformatics analysis, which requires more experimental validation in future.

## Conclusions

In summary, we identified that SERPINE1, the key regulator of angiogenesis and the uPA system, was involved in gastric tumorigenesis. Consistent with previous reports, we discovered that SERPINE1 was overexpressed in GC tissues and correlated with a poor prognosis in GC. Further study revealed the overexpression of SERPINE1 in the EMT subtype of GC, and GSEA analysis confirmed that gene sets involved in EMT were significantly enriched in GC patients with higher levels of SERPINE1. Correlation analysis and experimental validation identified significant correlations between the SERPINE1 and EMT markers in GC. The PPI network showed that FN1, TIMP1, SPARC, and MMP2 were closely connected with SERPINE1. Further investigations on potential mechanisms of SERPINE1 participating in EMT of GC are required. In conclusion, our study identified the involvement of SERPINE1 in EMT and potent correlations between SERPINE1 and EMT-related genes, which proved to be potential biomarkers and therapeutic targets in GC.

## Supplemental Information

10.7717/peerj.7091/supp-1 Supplemental Information 1 Raw data extracted from GSE54129 by applying GEO2R tool.

10.7717/peerj.7091/supp-2 Supplemental Information 2 Raw data extracted from GSE63089 by applying GEO2R tool.

10.7717/peerj.7091/supp-3 Supplemental Information 3 Raw data extracted from GSE65801 by applying GEO2R tool.

10.7717/peerj.7091/supp-4 Supplemental Information 4 Raw data of qRT-PCR validation.

## Additional Information and Declarations

### Data Availability

The following information was supplied regarding data availability: The data is available at the Gene Expression Omnibus(GEO) database: GSE54129, GSE63089, GSE65801.
